# Supplementary material for: Comparative stability analysis of Indonesian banks: Markov Switching—Dynamic Regression for Islamic and conventional sectors
Source: PLoS One. 2024 Apr 18;19(4):e0301398. doi: 10.1371/journal.pone.0301398 (PMC11025910; doi:10.1371/journal.pone.0301398)
Supplement: Attachment — Submitted filename: IMRON_Response to Reviewers_EWS.pdf [file pone.0301398.s001.pdf]

Response Table to Reviewer Suggestions on Manuscript Comparative Stability Analysis of Indonesian Banks: Markov Switching—Dynamic Regression for Islamic and Conventional Sectors (PONE-D-23-38458)

No.	Comments	Respons
1	The article focuses on identifying the factors influencing the stability and crisis impact of Islamic Commercial Banks (ICB) and Islamic Rural Banks (IRB), and compares the stability of the Islamic banking industry and the traditional banking industry in Indonesia under different economic systems (crisis and stability). This is important for understanding the dynamics that affect the stability of the Islamic and traditional banking industries in the Indonesian context.<br>The main focus of the article is on the systemic risk of Islamic Commercial Banks (ICB) and Islamic Rural Banks (IRB) in the Indonesian context, which has a unique cultural background.<br><br>However, in the fourth section "4. Results and Analysis," **why is it necessary to study the impact of the Financing to Deposit Ratio (FDR) on the Industrial and Commercial Bank of China and the China Construction Bank?**<br><br>**The banking systems of China and Indonesia are products of different economic systems, so what is the significance of this part of the research?** Please provide a detailed explanation and clarification.	Thanks for your comment.<br>We would like to clarify that CCB in this manuscript is an acronym for Conventional Commercial Bank, not Commercial Bank of China or China Construction Bank.<br><br>The reason we discuss FDR in this research is to determine bank liquidity conditions. Bank liquidity is important to know because it is a measure of sufficient reserves for customers to withdraw funds. We have explained this on Page 13, last paragraph.
2	This is a very interesting paper describing comparative stability analysis of Indonesian Banks, using the Markov Switching -Dynamic Regression. But there are some queestions needed to revise to smooth the paper.<br><br>First, the logic and format of the paper is not good, and some paragraph is repetitive. So, **the author need to reorganize the paragraph and read carefully to make the logic clear and form a story, such as the table should be three-wire meter**. Because the paragraph is now dissected.<br><br>Second, many tables showed in the paper with different categories, so the **the author should unify to one table, such as table 1 and table 2 can be merge into one table,** and **the decimal places of the table should be united, which is about three decimal palces**.<br><br>Third, the author **should make the Zt explaination of the equation 1**, which is not existed in page 12.	Thank you for your advice. To improve the logic clear and form a story, we have changed the draft in the INTRODUCTION and METHODOLOGY sections. Please kindly review these sections.<br><br>Following the reviewer's advice, we merged several tables. The list of tables that we merge includes:<br>Tables 1 and 2 become Table 1<br>Tables 3 – 6 become Table 2<br>Tables 7 and 8 become Table 3<br>Tables 9 and 10 become Table 4.

		We've also made all the decimal places of the table about three decimal places.<br><br>We have also made the Zt explanation of the equation 1 based on bank type and further differentiated based on regime classification. See Equations 2 – 6.
3	**Please ensure that your manuscript meets PLOS ONE's style requirements**, including those for file naming. The PLOS ONE style templates can be found at https://journals.plos.org/plosone/s/file?id=wjVg/PLOSOne_formatting_sample_main_body.pdf and https://journals.plos.org/plosone/s/file?id=ba62/PLOSOne_formatting_sample_title_authors_affiliations.pdf	We have changed our manuscript according to the PLOS ONE style templates
4	Thank you for stating the following financial disclosure<br><br>**Please state what role the funders took in the study.** If the funders had no role, please state: "The funders had no role in study design, data collection and analysis, decision to publish, or preparation of the manuscript."<br>If this statement is not correct you must amend it as needed.<br>**Please include this amended Role of Funder statement in your cover letter**; we will change the online submission form on your behalf.	We have included the role of the funders. See Funder Role Statement on Page 28, also on our cover letter.
5	We note that your Data Availability Statement is currently as follows: [All relevant data are within the manuscript and its Supporting Information files.]<br><br>Please confirm at this time whether or not your submission contains all raw data required to replicate the results of your study. **Authors must share the "minimal data set" for their submission**. PLOS defines the minimal data set to consist of the data required to replicate all study findings reported in the article, as well as related metadata and methods (https://journals.plos.org/plosone/s/data-availability#loc-minimal-data-set-definition).	Thank you for the advice. In the "Data Availability Statement" section, we state the data that supports the findings of this study are available publicly from the Indonesian Financial Service Authority (OJK), Bank Indonesia, and Indonesian Statistics without any restriction.<br>For more details, we provide an explanation in the Methods section on page 10, 2nd paragraph.
6	**If there are ethical or legal restrictions on sharing a de-identified data set, please explain them in detail** (e.g., data contain potentially sensitive information, data are owned by a third-party organization, etc.) and who has imposed them (e.g., an ethics committee). Please also provide contact information for a data access committee, ethics committee, or other institutional body to which data requests may be sent. If data are owned by a third party, please indicate how others may request data access.	Thank you for your reminder. There are No ethical or legal restrictions on using data sets that we used in this manuscript. We add the explanation on "Ethical Statement" section, See page 28.

7	**Please include your full ethics statement in the 'Methods' section of your manuscript file.** In your statement, please include the full name of the IRB or ethics committee who approved or waived your study, as well as whether or not you obtained informed written or verbal consent. If consent was waived for your study, please include this information in your statement as well.	This research does not include humans or animals as research objects.<br>We use secondary data from third parties (Financial Service Authority (OJK), Bank Indonesia, and Indonesian Statistics) which can be used without prior permission. We have explained this on the "Data Availability Statement" and "Ethical Statement". See page 28.
